# Fungal Microbiota Dysbiosis and Ecological Alterations in Gastric Cancer

**DOI:** 10.3389/fmicb.2022.889694

**Published:** 2022-04-29

**Authors:** Ping Yang, Xiaoshan Zhang, Rui Xu, Khan Adeel, Xiaofeng Lu, Min Chen, Han Shen, Zhiyang Li, Zhipeng Xu

**Affiliations:** ^1^Department of Clinical Laboratory, The Affiliated Drum Tower Hospital of Nanjing University Medical School, Nanjing, China; ^2^College of Life Science, Yangtze University, Jingzhou, China; ^3^State Key Laboratory of Pharmaceutical Biotechnology, Department of Physiology, Jiangsu Engineering Research Center for MicroRNA Biology and Biotechnology, NanJing Universit Advanced Institute of Life Sciences (NAILS), School of Life Sciences, Nanjing University, Nanjing, China; ^4^State Key Laboratory of Bioelectronics, National Demonstration Center for Experimental Biomedical Engineering Education, School of Biological Science and Medical Engineering, Southeast University, Nanjing, China; ^5^Department of Gastrointestinal Surgery, The Affiliated Drum Tower Hospital of Nanjing University Medical School, Nanjing, China; ^6^Department of Gastroenterology, The Affiliated Drum Tower Hospital of Nanjing University Medical School, Nanjing, China; ^7^Jiangsu Province Key Laboratory of Modern Pathogen Biology, Department of Pathogen Biology, Nanjing Medical University, Nanjing, China

**Keywords:** fungal microbiota, gastric cancer, pathogen, inflammation, biomarker

## Abstract

Changes in bacteriome composition have a strong association with gastric cancer (GC). However, the relationship between stomach fungal microbiota composition and human host immune factors remains largely unknown. With high-throughput internal transcribed spacer region 2 (ITS2) sequencing, we characterized gastric fungal microbiome among the GC (*n* = 22), matched para-GC (*n* = 22), and healthy individuals (*n* = 11). A total of 4.5 million valid tags were generated and stratified into 1,631 operational taxonomic units (OTUs), and 10 phyla and 301 genera were identified. The presence of GC was associated with a distinct gastric fungal mycobiome signature, characterized by a decreased biodiversity and richness and significant differences in fungal composition. In addition, fungal dysbiosis was reflected by the increased ratio of *Basidiomycota* to *Ascomycota* and a higher proportion of opportunistic fungi, such as *Cutaneotrichosporon* and *Malassezia*, as well as the loss of *Rhizopus* and *Rhodotorula* during the progression of cancers. A panel of GC-associated fungi (e.g., *Cutaneotrichosporon* and *Rhodotorula*) was found to adequately exhibit diagnostic value. Furthermore, the mRNA levels of cytokines and chemokines were detected and correlated with the specific fungal dysbiosis, indicating the possible mechanism of GC. This study reveals GC-associated mycobiome dysbiosis characterized by altered fungal composition and ecology and suggests that the fungal mycobiome might play a role in the pathogenesis of GC.

## Introduction

Gastric cancer (GC) is a dangerous disease and in 2020, it ranked 3rd in causing human deaths around the world, and it is the fifth most widely diagnosed cancer (Ferlay et al., 2021). GC typically develops through multisteps, from atrophic gastritis (AG) that progresses into intestinal metaplasia (IM) eventually manifesting as GC. Host–microbiota interaction, such as gastric microbial infections and host immune factors, has been shown to be contributing to tumor development in gastric system (Dicken et al., 2005). However, the etiology and pathogenesis, as well as their translational roles in the pathogenesis of GC, need further research for efficient elucidation.

Recent evidence indicates the involvement of the gastric microbiome in the disease onset and progression. The stomach was considered “a hostile place” for bacterial growth since the conditions were unsuitable for microbial growth (Rajilic-Stojanovic et al., 2020). Since the 1980s, *Helicobacter pylori* was found to be the most common microbial for gastrointestinal (GI) tract disorders (Parkin et al., 2005; Polk and Peek, 2010; de Sablet et al., 2011). However, among all the patients infected with *H. pylori*, only 1–3% will finally develop GC (Wang et al., 2014), and the progression of cancers can still be discovered after eradication of *H. pylori* by pharmacological treatment (Fukase et al., 2008), indicating that not only *H. pylori* but other microbes can also parasitize in the stomach and have a role in GC onset and progression.

Advances in high-throughput sequencing technologies made it possible to find the alterations of gastric microbial composition and characteristics in healthy and illness stages (Dong et al., 2019; Deng et al., 2021; Yang et al., 2021). A study has provided evidence that the oral bacteria were more likely to aggregate in the GC samples (Chen et al., 2019), and the species *Streptococcus, Prevotella, Neisseria, Haemophilus*, and *Porphyromonas* were provided to be the most dominant bacteria in GC (Bik et al., 2006). The emerging or re-emerging fungal is becoming a worldwide public health threat closely associated with the immune modulation of the host (Lockhart and Guarner, 2019). Recent studies have confirmed the fungal compositional alterations in colorectal adenoma tissue (Coker et al., 2019), Crohn's disease (Liguori et al., 2016), and patients with ulcerative colitis (Sokol et al., 2017), providing opportunities for the discovery of a new relationship between host and fungal microbiome interactions. Relatively, there is a paucity of research to better understand the functional role of gastric fungal microbiota in GC, especially from the perspective of their potential diagnostic value in the screening of GC.

In this study, through a comprehensive analysis of the fungal microbiomes in GC, we aimed to highlight and understand various components of the gastric fungal microbiome; signify the clinical relevance of specific fungi that can be crucial for GC pathogenesis and diagnosis that can be harnessed to develop better prevention and treatment strategies for GC. To the best of our knowledge, this study is the first of its kind that provides detailed evidence for the interactions between host immune factors and fungal microbiome, especially for the potential diagnostic significance of fungi in GC.

## Materials and Methods

### Research Design and Sample Collection

**Figure 1:**
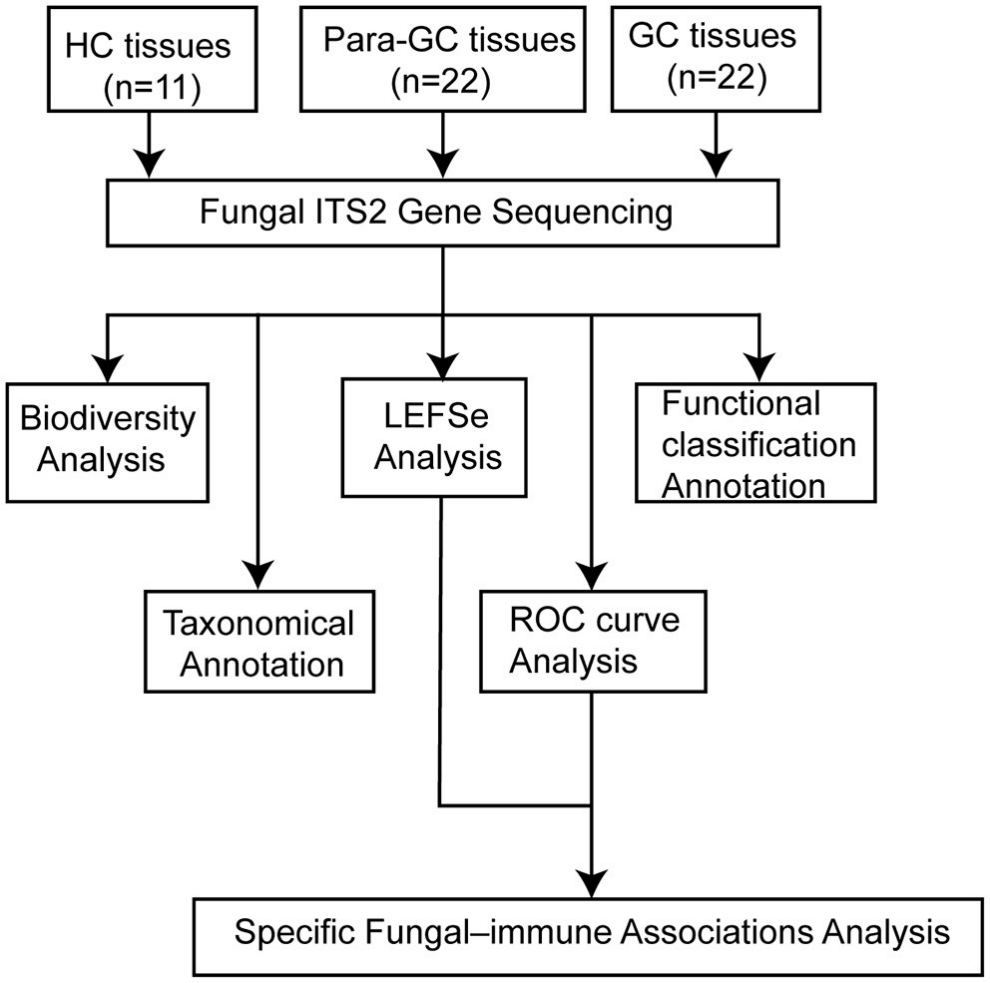
This flowchart illustrates the conceptual framework for the proposed study. We totally enrolled 11 healthy controls (HC) and 22 patients with gastric cancer (GC), collecting the tissues for fungal internal transcribed spacer region 2 gene sequencing. Then, we compared the biodiversity among the three groups, obtained the taxonomic information at the phylum and genus level, used the linear discriminant analysis (LDA) effect size (LEFSe) and receiver operating characteristic (ROC) curve analysis to identify the different fungi, measured the relative mRNA levels of inflammatory factors, and performed the specific fungal-immune association analysis to find out the possible mechanism of fungal in GC. Finally, we used the FUNGuild to further predict the functional classification of the specific fungal community.

We enrolled 22 patients with GC confirmed by surgery combined with pathologic biopsy and 11 healthy controls (HC) from the Affiliated Drum Tower Hospital of Nanjing University Medical School in this study. A total of 22 GC tissues and 22 para-GC tissues collected by surgery and 11 HC tissues collected by endoscopic biopsy were selected for microbiota analysis based on the American Society for Gastrointestinal Endoscopy guideline (ASGE Standards of Practice Committee et al., 2013) (Figure 1). The GC tissue we collected was obtained by total or partial gastrectomy. Location of tumor resection specimen includes antrum, body, and cardia. The adjacent tissue we selected is the tissue around the cancer tissue, which is 2 cm away from the cancer tissue. The inclusion criteria of the healthy group were healthy patients who came to the hospital for physical examination, without tumors, diabetes, and other digestive diseases. Ethics Committee of the Affiliated Drum Tower Hospital of Nanjing University Medical School approval was obtained for the study (ID: 2021-514-02). From every participant of the study, informed written consent was also obtained.

### PCR Amplification

For subsequent examination, all specimens were kept at −80°C. DNA was extracted using the E.Z.N.A.® soil DNA Kit (Omega Bio-Tek, Norcross, GA, USA) according to the manufacturer's guidelines. Fungal internal transcribed spacer region 2 (ITS2) amplification was performed using the primers ITS3F GCATCGATGAAGAACGCAGC and ITS4R TCCTCCGCTTATTGATATGC. The DNA content and cleanliness were tested using a NanoDrop 2000 UV-vis spectrophotometer, and the DNA samples were validated on a 1% agarose gel (Thermo FisherScientific, Wilmington, USA).

The ITS2 rRNA PCR amplification was performed using the following protocol: initial denaturation at 95°C for 3 min, followed by 35 cycles of denaturing at 95°C for 30 s, annealing at 55°C for 30 s, and extension at 72°C for 45 s, then single extension at 72°C for 10 min, and 10°C until stopped. Of note, 2 × pro Taq buffer 10 μl, forward primer (5 μM) 0.8 μl, reverse primer (5 μM) 0.8 μl, template DNA 20 ng/μl, and last ddH2O up to 20 μl were used to make up the PCR mixes. Triplicate PCR reactions were carried out. The PCR product was purified and quantified using the AxyPrep DNA Gel Extraction Kit (Axygen Biosciences, Union City, CA, USA) according to the manufacturer's instructions (Promega, USA).

Using an Illumina MiSeq platform, extracted amplicons were aggregated in an equimolar ratio and paired-end sequencing (Illumina, San Diego, USA). The raw reads were deposited in the Sequence Read Archive (SRA) database at the National Center for Biotechnology Information (NCBI) (Accession Code: PRJNA797736).

### Processing of Sequencing Results and Taxonomical Annotation

The raw ITS2 rRNA gene sequencing reads were demultiplexed, quality-filtered by FASTP version 0.20.0 (Chen et al., 2018), and merged by FLASH version 1.2.7 (Magoc and Salzberg, 2011) with the following criteria: (i) the 300 bp reads were truncated at any site receiving an average quality score of <20 over a 50 bp sliding window, the truncated reads shorter than 50 bp were discarded, and reads containing ambiguous characters were also discarded; and (ii) only overlapping sequences longer than 10 bp were assembled according to their overlapped sequence. The maximum mismatch ratio of the overlap region is 0.2. Reads that could not be assembled were discarded. (iii) Samples were identified based on barcodes and primers at both ends of the sequence, and the sequence direction was adjusted, exact barcode matching, 2 nucleotides mismatches in primer matching. Operational taxonomic units (OTUs) with a 97% similarity cutoff value (Edgar, 2013) were clustered into the same operational classification unit using the UPARSE version 7.1, and chimeric sequences were identified and removed. Based on sequencing accuracy, alpha diversity was calculated with QIIME, including an index of observed_species, chao1, and PD_whole_tree (Schloss et al., 2009). PCoA of the Bray–Curtis distance with each sample colored by the disease phenotype was built and used to assess the variation between experimental groups. The taxonomical classification of fungal OTUs was performed according to the UNITE (Release 8.2) databases (Koljalg et al., 2013).

### Total Tissue RNA Extraction and Quantitative RT-PCR for Cytokines mRNA

Through the use of an RNA extraction kit (Invitrogen), total RNA was retrieved from tissues (retained at −80°C) and reversed according to the reverse transcription kit's instructions (Vazyme). The 2^−Δ*ΔCt*^ technique had been used to calculate the relative mRNA level of cytokines considering *GAPDH* as an internal reference. Supplementary Table 1 lists the primers.

### Statistical Analysis

Biomarker discovery analysis was performed by linear discriminant analysis (LDA) effect size (LEfSe) (Segata et al., 2011) combined with the Kruskal–Wallis rank-sum test to detect the features with significantly different abundances between assigned taxa and to estimate the effect size of each feature. The SPSS 24.0 statistical software had been used to run a two-tailed Mann–Whitney *U* test, and GraphPad Prism 8.0 (GraphPad, San Diego, CA, USA) had been used to determine statistically significant differences among case and control groups. The Wilcoxon rank test, Turkey group test, permutational multivariate analysis of variance (PERMANOVA), and Random Forest analysis were performed by the R project, and the fungal functional classification prediction was inferred by using FUNGuild (Nguyen et al., 2016) (version 1.0). The Gini index is calculated by python and plotted by ggplot.

## Results

### Basic Information of Study Participants

In this study, we collected 55 tissues from 11 HCs and 22 patients with GC at the Affiliated Drum Tower Hospital of Nanjing University Medical School, dividing them into three groups (i.e., 11 HCs, 22 GC, and 22 para-GC) for the sake of comparability. The detailed clinical characteristics are shown in Table 1; Supplementary Table 2. The information of participants contains age, gender, and some clinical biochemistry indices. GC and HC have no significant differences in age. Furthermore, four serum tumor biomarkers (i.e., AFP, CEA, CA-125, and CA-199) in the digestive system seem to be unable to discern between controls and GCs.

**Table 1:** Demographic and clinical details of samples.

**Index**	**Gastric carcinoma** **(*n* = 22)**	**Healthy control** **(*n* = 11)**	***P*-value** (*P <0.05)
Gender (Female, %)	6 (27.27%)	7 (63.64%)	
Age	60.59 ± 12.73	52.64 ± 10.92	0.0866
PG-I (ng/mL)	80.60 ± 52.66	40.35 ± 13.48	0.0189*
PG-II (ng/mL)	17.70 ± 9.98	7.87 ± 4.73	0.0043*
PGR (PG- I/ PG-II)	4.75 ± 1.66	5.71 ± 1.63	0.1224
AFP (ng/mL)	49.62 ± 212.38	2.33 ± 1.76	0.4693
CEA (ng/mL)	3.92 ± 5.51	1.17 ± 0.66	0.1121
CA-125 (U/mL)	7.89 ± 4.74	8.20 ± 2.55	0.9283
CA-199 (U/mL)	38.01 ± 75.898	10.94 ± 7.14	0.3595
ALT (U/L)	16.05 ± 11.46	18.05 ± 8.76	0.6143
AST (U/L)	16.91 ± 6.22	18.82 ± 4.52	0.3737
Total bilirubin (μmol/L)	8.76 ± 3.71	12.97 ± 3.68	0.0206*
Total protein (g/L)	58.77 ± 12.76	73.42 ± 4.31	0.0110*
Globulin (g/L)	22.83 ± 3.41	28.97 ± 3.31	0.0006*
Albumin to globulin ratio	1.72 ± 0.28	1.56 ± 0.17	0.2818
Total bile acid (μmol/L)	7.26 ± 8.018	1.93 ± 0.79	0.2030
Glucose (mmol/L)	5.03 ± 1.44	5.03 ± 0.51	0.9928
Urea (mmol/L)	5.77 ± 2.12	5.52 ± 0.99	0.7098
Creatinine (μmol/L)	69.50 ± 17.22	65.36 ± 11.24	0.4765
Uric acid (μmol/L)	302.45 ± 77.20	318.73 ± 58.90	0.5349
Triglycerides (mmol/L)	1.58 ± 1.06	1.05 ± 0.54	0.1318
Cholesterol (mmol/L)	4.18 ± 0.77	5.24 ± 0.61	0.0004*
H-cholesterol (mmol/L)	1.13 ± 0.31	4.59 ± 0.39	0.0009*
L-cholesterol (mmol/L)	2.41 ± 0.67	3.11 ± 0.48	0.0043*
CRP (mg/L)	4.26 ± 2.03	4.45 ± 4.98	0.8933

### Comparison of the Microbial Diversity Between Patients With GC and HC

We performed the ITS2 gene sequencing on the tissues to assess the composition of the fungal microbiota among the three groups. The sequencing yielded more than 4.5 million tags, with the dominant length of tags located among 260–320 bp after trimming and filtering (Supplementary Table 3). According to different similarity levels, all sequences were divided into 1,761 OTUs and 1,631 OTUs were finally obtained after specific processing. To evaluate the sequencing depth of all groups, the goods coverage index was selected, and all groups are >0.990. The rarefaction curves in both the GC and control samples tended to plateau, indicating that our sequencing depth and coverage were satisfactory and implicitly mirrored the species richness (Supplementary Figure 1).

**Figure 2:**
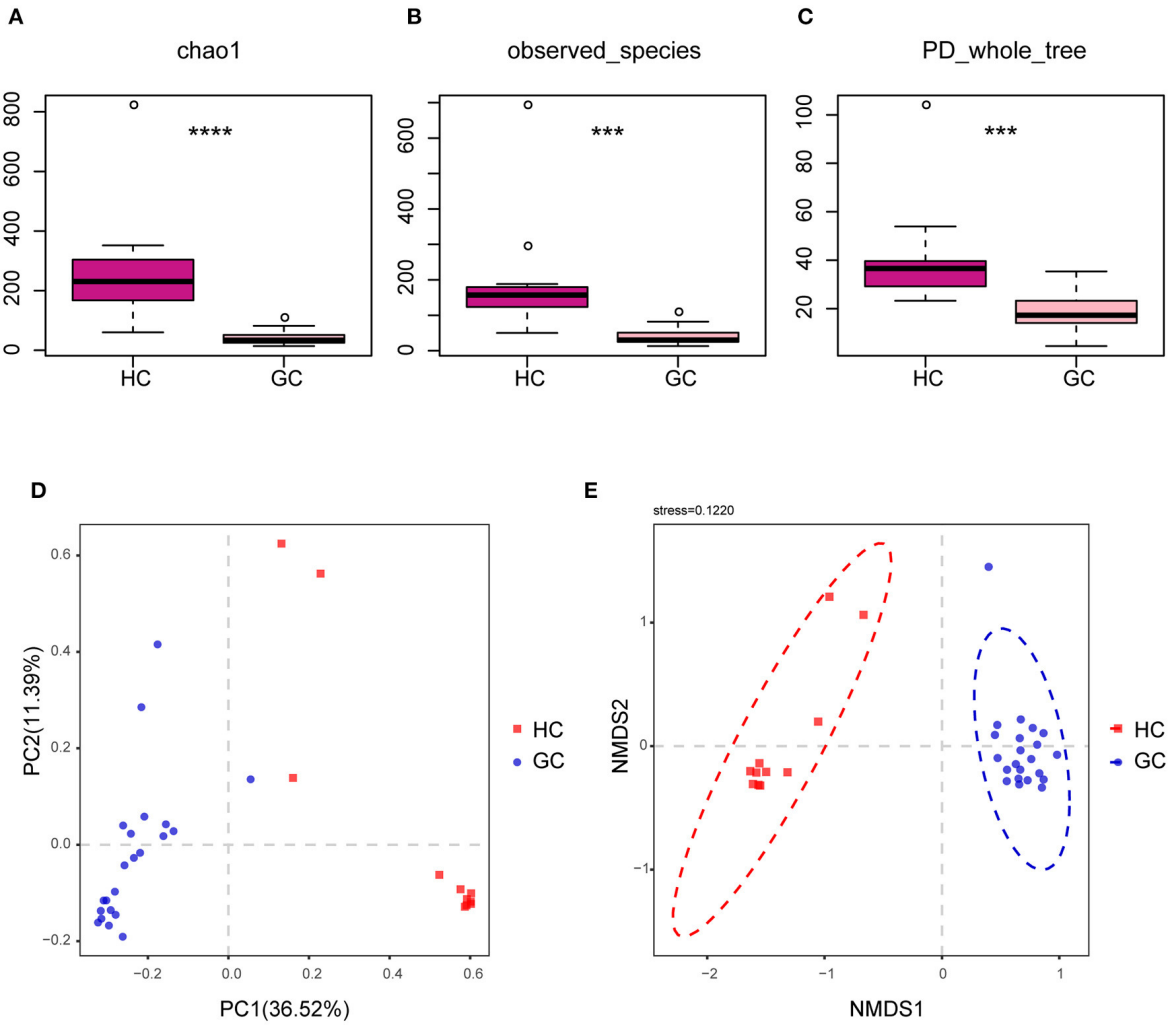
Altered bacterial microbiota biodiversity in GC. **(A–C)** Alpha diversity. Chao1, observed_species, and PD_whole_tree describe the alpha diversity of the fungi in GC and HC groups (Tukey test, ***p < 0.001; ****p < 0.0001). **(D,E)** Principal coordinate analysis of Bray–Curtis distance with each sample colored according to different groups. PC1 and PC2 represent the top two principal coordinates that captured most of the diversity. The fraction of diversity captured by the coordinate is given as a percentage. Groups were compared using the PERMANOVA method.

We looked at the alpha and beta diversities of fungal fractions to see whether our reference database was of good quality. Alpha diversity, a measure of genera richness (number of genera), was evaluated by chao1, observed_species, and PD_Whole_tree. Results in Supplementary Figures 2A–C indicate no significant difference in alpha diversity between the GC and the matched para-GC groups (chao1: *p* > 0.05; observed_species: *p* > 0.05; PD_Whole_tree: *p* > 0.05). The site-by-site alpha diversity analysis shows a peculiar variation with the lowest diversity in the GC tissues than the normal HC (chao1: *p* < 0.0001; observed_species: *p* = 0.0001501471; PD_whole_tree: *p* = 0.0002492035) (Figures 2A–C), demonstrating that microbial diversity and richness reduced in the creation and progression of GC.

The beta diversity was next used to assess the microbial community compositions among the three groups by performing PERMANOVA with Bray–Curtis distance, which accounts for both patterns of presence–absence of taxa and changes in their relative abundance. The PCoA showed that individuals belonging to GC groups formed fairly well-separated from the HC (Figure 2D). In addition, the nonparametric multidimensional scaling (NMDS) is applied to visualize the distances between categories, as shown in Figure 2E, illustrating the distinct separation between the GC tissues and HC samples. However, there were no significant distinct groups between GC and para-GC groups, which essentially overlapped (Supplementary Figures 2D,E). Altogether, these results reveal that the GC group exhibited more unique fungal profiles than the HC group. Considering the number of samples, stages I and II are defined as early GC, and stages III and IV are defined as advanced GC. Results showed no significant difference in fungi between early and advanced GC (Supplementary Figure 3).

### The Gastric Fungal Profile Differs in Patients With GC

**Figure 3:**
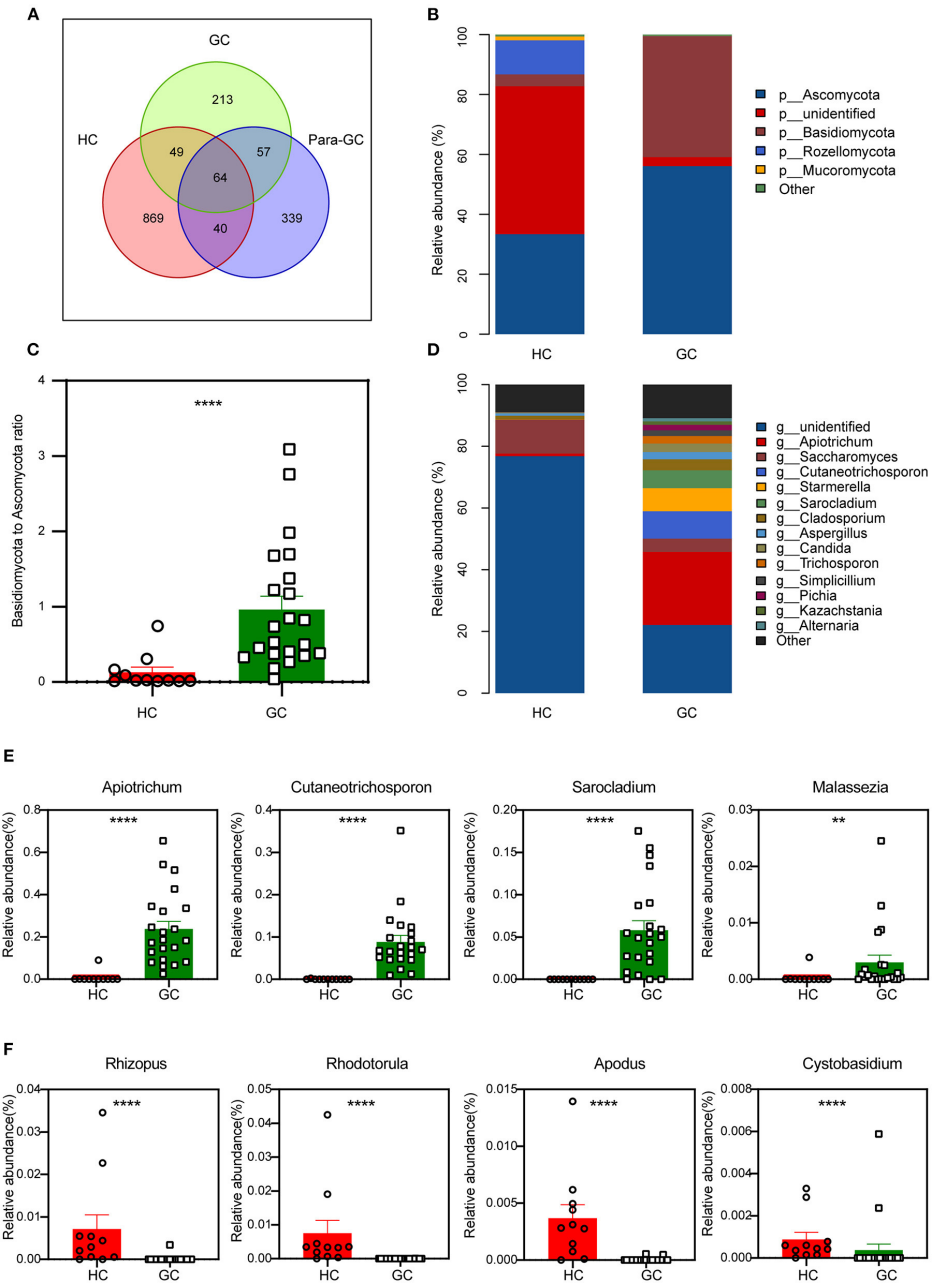
Changes of the fungal composition. **(A)** Venn diagram analysis according to the operational taxonomic unit abundance among the three groups. **(B)** Comparisons of the relative abundance of dominant fungal taxa at the phylum level, “p” represents phylum. **(C)** The ratio of *Basidiomycota* to *Ascomycota* relative abundance between the two groups (Mann-Whitney *U* test, ****p < 0.0001). **(D)** Comparisons of the relative abundance of dominant fungal taxa at the genus level, “g” represents genus. **(E,F)** The differentially abundant fungal genus of GC-enriched **(E)** and HC-enriched **(F)** between HC and GC groups (Mann-Whitney *U* test, **p < 0.01; ****p < 0.0001).

We next used a Venn diagram to show the distribution of common and endemic OTUs according to the OTUs abundance. There were 64 OTUs that were shared among the three groups, with 869,213 and 339 OTUs unique for the HC, GC, and para-GC groups, respectively (Figure 3A). The counts of OTUs in the HC group were 4-fold higher than the GC group and 3-fold higher than the para-GC group, revealing that the fungi of GC were different from those of normal controls.

We matched the OTU representative sequences with the Unite database to acquire taxonomic information for the OTUs from phylum to species level based on these findings. In particular, there were considerable variations in abundance between both the HC and GC groups at the phylum level, but no statistical differences between the GC and para-GC groups (Supplementary Figure 4). Compared with the HC group, GC had a dominant abundance of the *Ascomycota*, followed by *Basidiomycota* (Figure 3B). A dramatically declined abundance of *Rozellomycota* and an obvious increasing tendency of *Basidiomycota* and *Ascomycota* can also be found in GC compared with the HC group. Moreover, the ratio of *Basidiomycota* to *Ascomycota*, which has been reported that can reflect fungal dysbiosis (Coker et al., 2019), was higher in GC than in HC group (Figure 3C, *p* < 0.0001). Additional differences were observed at lower taxonomic levels, the healthy gastric fungal was composed of *Saccharomyces* (11.0%), *Cladosporium* (1.3%), and the unidentified fungal genus contributed to the vast majority (76.9%). This result is consistent with a previous study, which confirmed that the most prevalent fungal genus in healthy individuals contains *Saccharomyces* and *Cladosporium* (26). Additionally, *Apiotrichum* was found to be the dominant genus of the GC groups (23.7%), followed by *Cutaneotrichosporon* (8.8%), *Starmerella* (7.5%), *Sarocladium* (5.8%), *Saccharomyces* (4.3%), and *Cladosporium* (3.6%) (Figure 3D). Furthermore, the proportion of several fungal were significantly enriched in the GC group, including *Apiotrichum* (*p* < 0.0001), *Cutaneotrichosporon* (*p* < 0.0001), *Sarocladium* (*p* < 0.0001) as well as *Malassezia* (*p* = 0.004) (Figure 3E). *Rhizopus* (*p* < 0.0001), *Rhodotorula* (*p* < 0.0001), *Apodus* (*p* < 0.0001) and *Cystobasidium* (*p* < 0.0001) were seen to be enriched in HC groups (Figure 3F). Overall, the data points to the dysbiosis of the stomach's fungal microbiota, which might be linked to gastric carcinogenesis.

### Specific Taxonomic Changes

**Figure 4:**
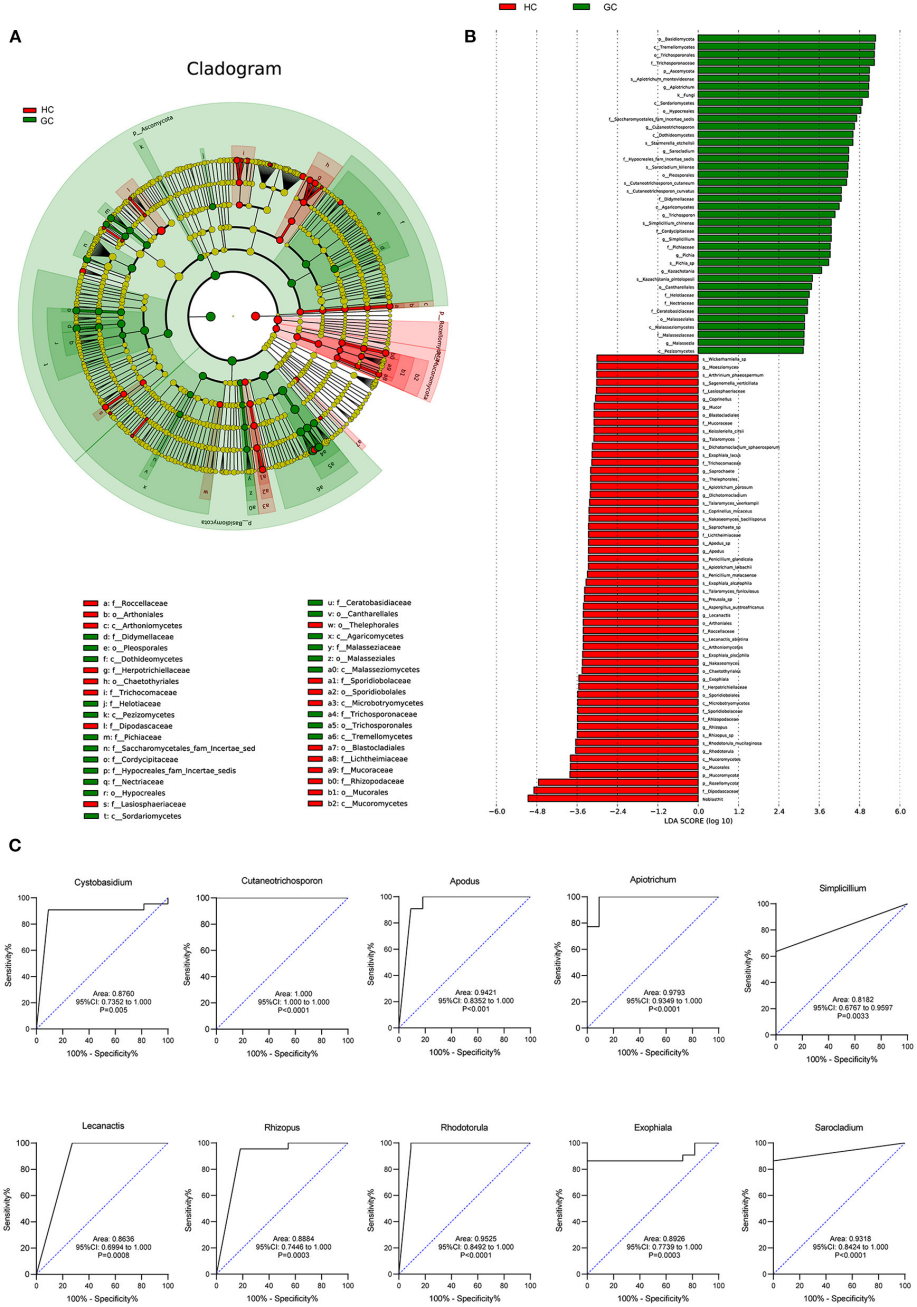
Fungal species as GC diagnostic markers. **(A)** Cladogram results for the different fungal taxa. Differences were represented by the color of the most abundant class (red, HC; green, GC; yellow, no significance). The diameter of each circle is proportional to the taxon's abundance. Each ring represents the next lower taxonomic level. **(B)** LDA score computed by LEfSe analysis between HC and GC groups (LDA score >3, red, HC; green, GC). **(C)** ROC curves of the top 10 fungal genus. Diagnostic performance for GC represented by the area under curve was shown.

To deeply characterize the microbiota alterations in GC, the multilevel LEfSe analysis was utilized for the groups in all taxa (Figures 4A,B). By comparison, the cladogram showed a total of 39 differential taxa, including nine classes, 11 orders, and 19 families, and was identified to be responsible for discriminating GC and HC groups (LEfSe: *p* < 0.05, *q* < 0.05, LDA > 3.0). The preponderance of fungal species at several levels was similar across the GC and HC groups. For example, *Malasseziaceae* at the family level, *Malasseziales* at the order level, and *Malasseziomycetes* at the class level enriched in GC, while *Sporidiobolaceae* at the family level, *Sporidiobolales* at the order level, and *Microbotryomycetes* at the class level aggregated in controls. LEfSe showed that 40 taxa enriched in the GC group, including *Apiotrichum, Cutaneotrichosporon, Sarocladium, Pichia, Chaetomium*, and *Malassezia*, and 58 taxa enriched in the HC group, containing *Rhodotorula, Exophiala, Rhizopus, Lecanactis, Coprinopsis, Russula*, and so forth. The details of other taxa are shown in Figures 4A,B.

### Fungal Microbiota-Based Prediction of GC

We further assessed the diagnostic ability of the top 10 microbial that displayed the most significant differences between GC and HC groups. The receiver operating characteristic (ROC) curves showed a great diagnostic potential, including *Cystobasidium* (area under curve [AUC] = 0.8760), *Cutaneotrichosporon* (AUC = 1.000), *Apodus* (AUC = 0.9421), *Apiotrichum* (AUC = 0.9793), *Simplicillium* (AUC = 0.8182), *Lecanactis* (AUC = 0.8636), *Rhizopus* (AUC = 0.8884), *Rhodotorula* (AUC = 0.9525), *Exophiala* (AUC = 0.8926), and *Sarocladium* (AUC = 0.9318) (Figure 4C). Combined with the Random Forest analysis (Supplementary Figure 5), these genera had an obvious effect in distinguishing GC and HC groups and thus can be used for diagnosis with a certain degree of accuracy.

### GC Microbiota Shows Specific Fungal-Immune Associations

**Figure 5:**
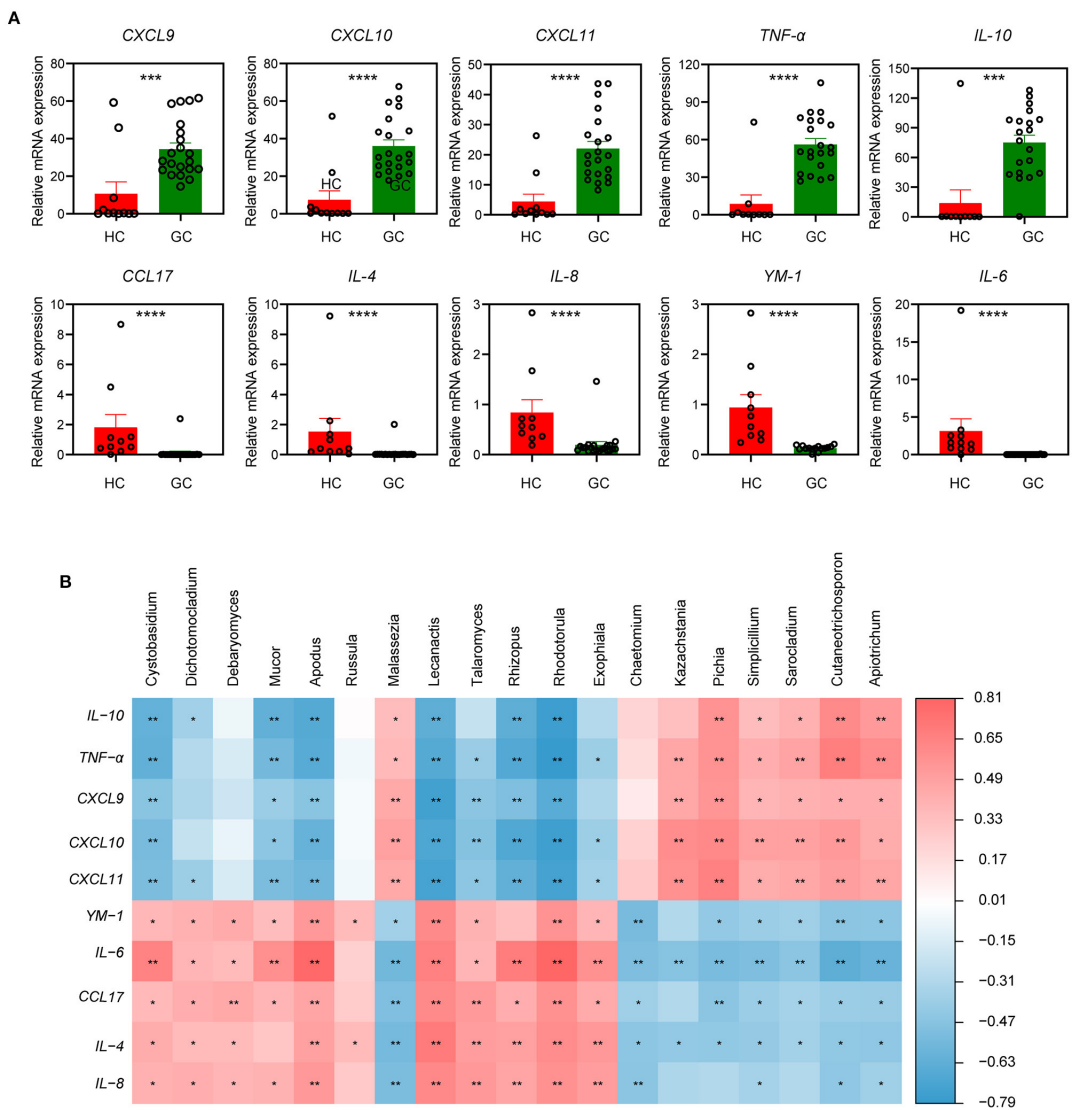
The specific relationships of fungal genus and immune-related factors. **(A)** The relative tissue mRNA level of 10 immune cytokines in HC and GC groups. The differences are calculated by Mann-Whitney *U* test (***p < 0.001; ****p < 0.0001). **(B)** Correlation analysis of the top 20 differential genera and 10 immune-related factors between the two groups according to the Spearman's correlation analysis. The correlation effect is indicated by a color gradient from blue (negative correlation) to red (positive correlation). Correlation coefficients and *p*-values (*p < 0.05; **p < 0.01) are shown.

Several polymorphisms of cytokines and chemokines have been implicated in cancer development from tumor initiation, promotion, and progression to metastasis, which may multiply the risk of carcinoma (Grivennikov et al., 2010; Tsujimoto et al., 2010; Lee et al., 2014). To further investigate the relationship of these functional factors and fungi between GC and HC groups, we then measured the relative mRNA levels of cytokine and chemokines in the tumor and normal tissue samples. The mRNA levels of pro-inflammatory cytokines and chemokines, such as *CXCL9, CXCL10, CXCL11*, and *TNF-*α, were markedly elevated in the GC group, whereas anti-inflammatory cytokines and chemokines, such as *CCL17, IL-4, IL-6, IL-8*, and *YM-1*, were dramatically lowered (Figure 5A). Interestingly, the GC group had a significantly higher amount of *IL-10* mRNA (Figure 5A), which is probably released by tumor-associated macrophages (TAMs) and creates an immune evasive microenvironment and dictates poor prognosis (Zhang et al., 2022).

It has also been reported that the mucosal levels of some cytokines were associated with *H. pylori* infection (Yamaoka et al., 1997), and a set of chemokines were involved in *H. pylori*-related immunopathologic responses (Jafarzadeh et al., 2019). We next performed a correlation analysis of the top 20 different fungi and immune-related factors to assess whether the fungal microbiota composition was associated with the disordered inflammatory response. Results in Figure 5B show a positive correlation between the GC-enriched fungi, such as *Apiotrichum, Cutaneotrichosporon, Simplicillium*, and *Sarocladium*, and several immune-related pro-inflammatory, such as *TNF-*α, *CXCL9, CXCL10*, and *CXCL11*, which implicated these four fungi, may participate in the tumor-promoting immune reaction. Furthermore, increased *IL-10* mRNA levels were positively linked with the presence of the four fungi stated earlier (Figures 5A,B). In contrast, the fungi reduced in GC containing *Cystobasidium, Apodus, Lecanactis, Rhizopus, Rhodotorula*, and *Exophiala* were positively correlated with immune factors that were characterized by anti-inflammatory properties (e.g., *IL-4, IL-6, YM-1*, and *CCL17*), suggesting that these genera have the potential to enhance anti-inflammatory response in GC. From above findings we can conclude that in gastric microbiota a dynamic interplay exists between fungi and immune related components and there are unique fungal alterations during GC.

### Functional Classification Prediction of the Specific Taxonomic

**Figure 6:**
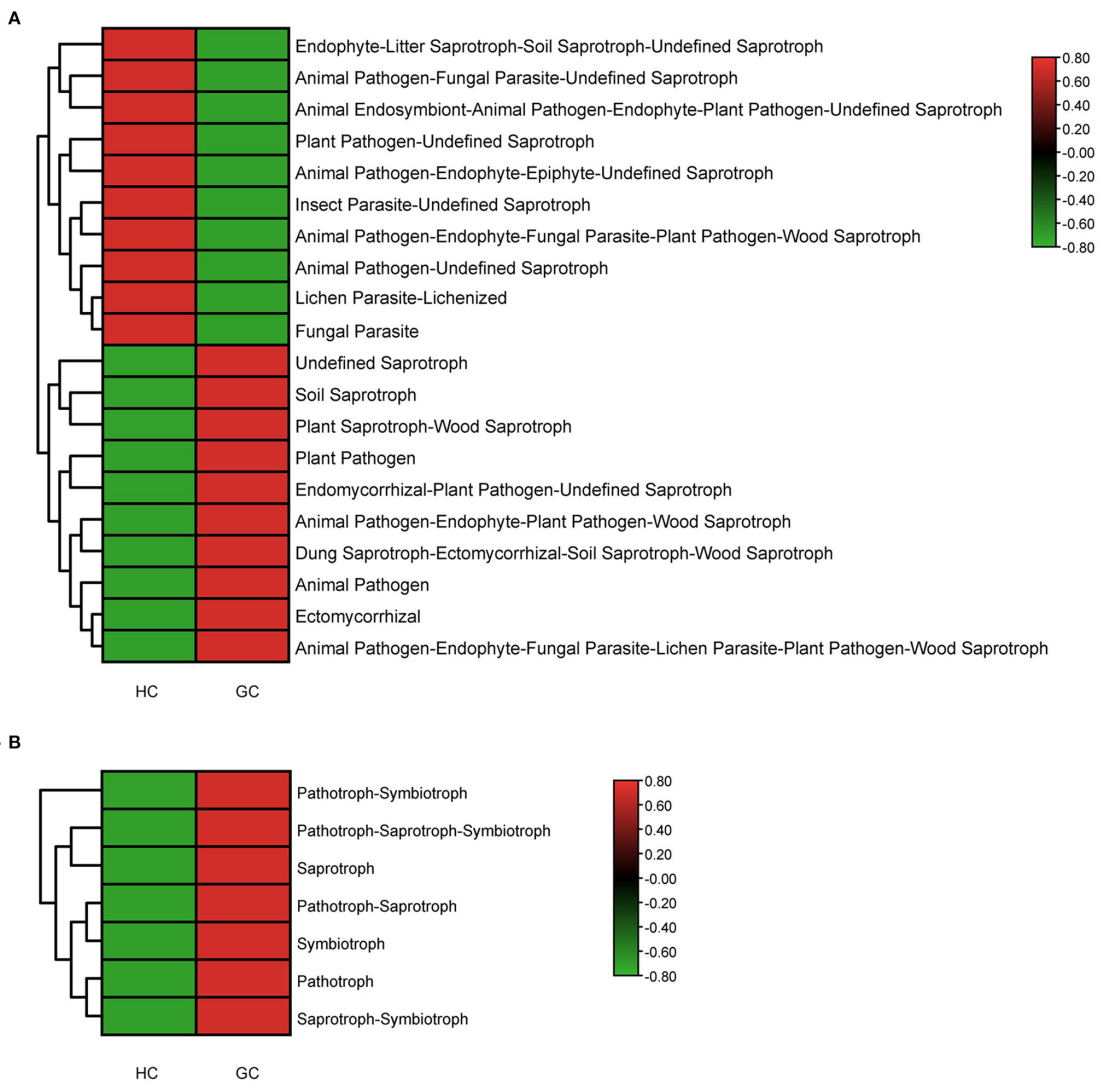
Functional classification predictions. Fungal functional annotations of the two groups were performed by FUNGuild. According to the ways in absorption and utilization of environmental resources, fungi were divided into different categories at the Guild **(A)** and Trophic **(B)** levels.

Due to the lack of a powerful instrument for annotating fungal function, we focused on the trophic modes and functional guilds of the fungal communities instead. We used FUNGuild to further predict the functional and nutritional classification of the specific fungal community. In the HC group, the most widely distributed functions are Undefined Saprotroph (64.3%), while in the GC group is Soil Saprotroph (54.9%). The most diverse guild between the two groups was Animal Pathogen. Besides, we used the heatmaps to describe the functional classification predictions by analyzing the Guild Sum and Trophic. There were distinctively differential functions between GC and HC groups (Figures 6A,B). Thus, the above results exhibit a unique symbiotic ecological relationship during the occurrence and development of GC, which is essential to maintain the gastric fungal homeostasis, and may provide a new strategy for further studies.

## Discussion

The fungal microbiome, which includes the *Fusarium* and *Aspergillus* genera as well as some other genera (e.g., *Alternaria* and *Mucor*) that make up the emerging pathogen group in humans, is increasingly acknowledged to play a significant role in cancer etiology (Perlin et al., 2017). The interaction of the fungal microbiota with the host has emerged as a critical factor in modifying host physiology and gastric microbiota-related diseases. In this article, we show that in GC, disease-specific alterations in the fungal microbiome persist. For the first time, we uncover the immunological process of GC pathogenesis and the predicted efficacy of fungi in patients with GC from the host–microbiota interaction perspective by investigating the fungal and local immune reactions concurrently.

With the advancement of the next-generation sequencing technologies, fungal taxa alterations have been proven to universally influence the development of host immune response and the initiation and progression of several cancers (Sokol et al., 2017; Aykut et al., 2019; Coker et al., 2019). Therefore, it is equally important to explore the relationship between the disorder of fungi and the exact mechanism underlying the development of GC. It is well-known that the microbiota associated with GI tissue or mucosa is not well-represented in paired stool specimens. In this study, we directly profiled the disease-specific fungal microbiome in GC tissue, which is often the only strategy to uncover specific dysregulated states associated with the diseased tissue microenvironments. Biodiversity was found to decrease in both GC samples and the paired para-GC samples, and the composition was modified with the reduction of *Rozellomycota* and increase of *Ascomycota* and *Basidiomycota* compared with the HC group. Besides, the ratio of *Basidiomycota* to *Ascomycota* and the proportion of opportunistic fungi, i.e., *Malassezia* and *Trichosporon*, which reflects fungal dysbiosis (Kazmierczak-Siedlecka et al., 2020), were higher in GC than HC groups. We also found that the gastric fungi discriminated GC and normal controls into two significantly distinct groups, indicating unique fungal profiles. Zhong et al. (2021) found that the abundance of *Fusicolla acetilerea, Arcopilus aureus*, and *Fusicolla aquaeductuum* was relatively high in GC tissue than in adjacent non-cancerous tissues, but no difference was observed between GC tissue and HCs, owing to the tight anatomical proximity and/or different population attributes in this investigation.

We also identified GC-specific shift in fungal composition, including nine classes, 11 orders, and 19 families, such as the enrichment of *Cutaneotrichosporon, Apiotrichum*, and *Malassezia* of the *Basidiomycota* phylum and the loss of *Lecanactis, Apodus*, and *Exophiala* of the *Ascomycota* phylum, implying that they may play a role in GC pathogenesis and necessitating further research. Previous studies have demonstrated that *Malassezia* usually exists in human skin and has the ability to colonize the GI tract (Richard et al., 2015; Sokol et al., 2017), and it can also interact with cells that are involved in immune functions and induce the production of a variety of cytokines and some secreted enzymes, eventually leading to carcinogenesis (Gaitanis et al., 2012). Interestingly, our results showed an obvious downregulation of *Rhodotorula* in GC, which is emerged as an opportunity for pathogens to infect susceptible patients, especially for cancers and AIDS (Miceli et al., 2011). Notably, we identified some previously unreported GC-associated fungi, which may be due to the different variables, such as area, age, gender, diet, and the sequencing methods that may affect microbiome compositions. We also evaluated the diagnostic value for the top 10 genera, all of the genera exhibited promising results. These observations provide an opportunity to apply distinctive fungi in detecting and monitoring the progression of GC. Undeniably, further study is required with a larger sample size, more clinical centers, and stricter screening criteria. To uncover the mechanism between dysregulated fungi and GC, further epidemiological research and bio-functional testing are recommended.

**Figure 7:**
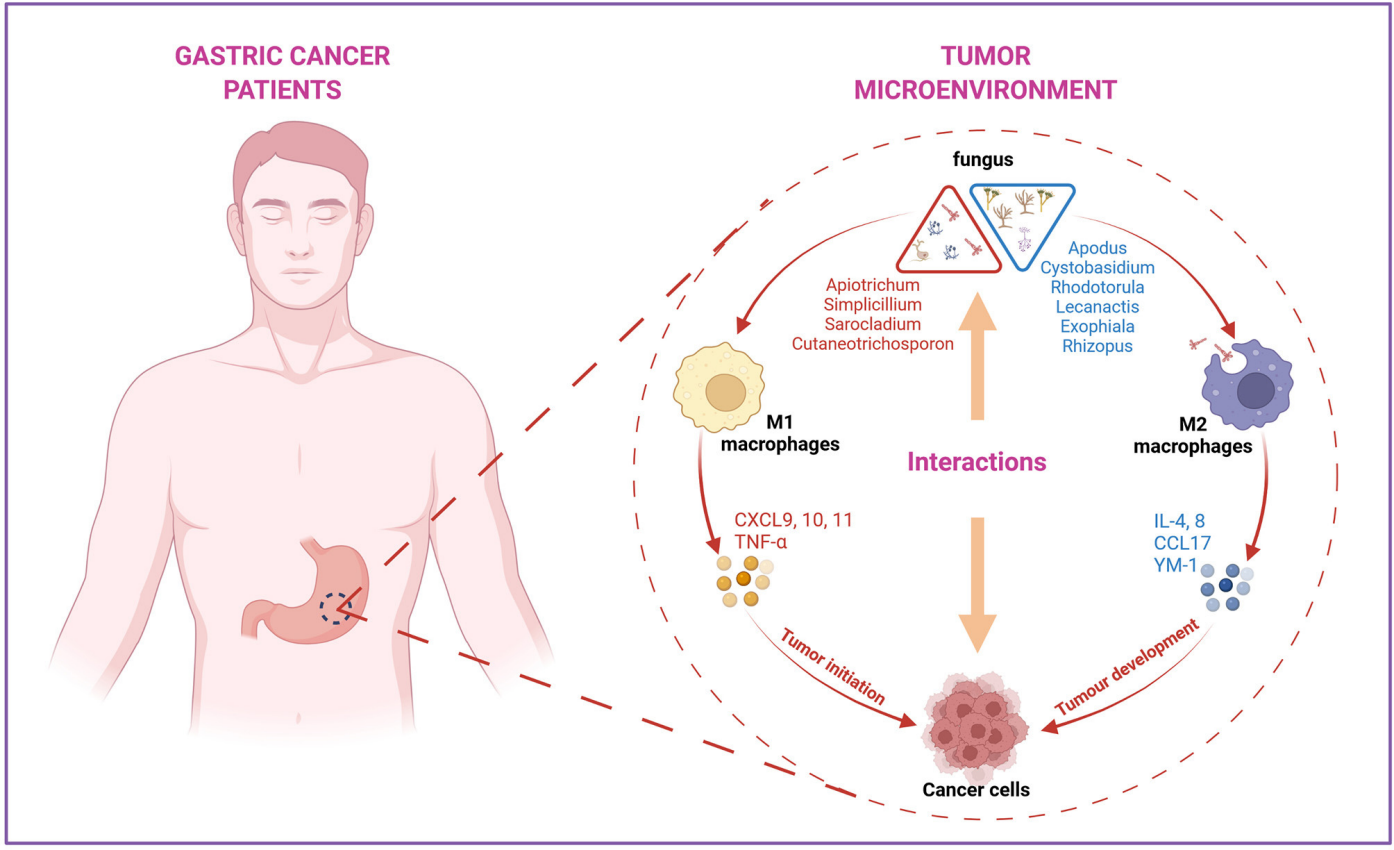
The summary of the interactions between specific fungus with immune factors in the tumor microenvironment during the progression of GC. *Apiotrichum, Simplicillium, Sarocladium*, and *Cutaneotrichosporon* were positively correlated with M1 macrophages-related pro-inflammatory factors, such as *CXCL9, 10, 11* and *TNF-*α, indicating these four fungi may involve in the initiation phase of tumor development. *Cystobasidium, Apodus, Lecanactis, Rhizopus, Rhodotorula*, and *Exophiala* were found to be positively correlated with M2 macrophages related anti-inflammatory factors *IL-6, IL-8, CCL17*, and *YM-1*, suggesting these genera may participate in the progression after tumor formation. Created with BioRender.com.

Aberrant cytokine production with the function of regulating angiogenesis, tumor growth, progression, and metastasis has been variously studied (Lee et al., 2014; Park et al., 2019). Pro-inflammatory chemokines *CXCL9, CXCL10*, and *CXCL11* were 2-fold overexpressed in GC compared to normal tissues (Lee et al., 2014), which is consistent with our data. *IL-10* was confirmed to be highly expressed in various types of cancer, including ovarian cancer (Ahmad et al., 2018), lymphoma (Gupta et al., 2012), prostate cancer (Lin and Zhang, 2017), and GC (Fortis et al., 1996), which can downregulate the inflammatory cytokines *IL-6* and *IL-8*. In our study, the mRNA levels of *IL-6* and *IL-8* were decreased in the GC group, probably due to the elevated *IL-10* level in the local area of the tumor. Interestingly, the associated analysis of different fungi in HC and GC groups indicated that immune responses were highly related to the variations in genera. The arguments of *CXCL9, CXCL10, CXCL11, TNF-*α, and *IL-10* are positively correlated with *Apiotrichum, Cutaneotrichosporon, Simplicillium*, and *Sarocladium*, while the downregulated *IL-4, IL-6, IL-8, CCL17*, and *YM-1* showed a positive association with *Cystobasidium, Apodus, Lecanactis, Rhizopus, Rhodotorula*, and *Exophiala*. All these results provide a basis for further investigation of the mechanisms of different fungal infections and GC (Figure 7).

The limitation of current evidence includes the modest size of tissues samples and the high scale of unclassified taxa. A larger cohort in multicenters and optimized sequencing methods are needed to validate our results in the future. Furthermore, our research indicates the prospective use of gastric fungal markers in the prediction of GC by describing the alteration of gastric fungal mycobiome homeostasis in GC carcinogenesis. We also discover changes in GC-specific fungal and immunological markers, indicating that synergistic host–fungi interactions may contribute to GC pathogenesis. To assist the development of therapeutic and diagnostic targets against GC, further research on the interactions between fungus, bacteria, and host is recommended.

## Data Availability Statement

The datasets presented in this study can be found in online repositories. The names of the repository/repositories and accession number(s) can be found below: https://www.ncbi.nlm.nih.gov/, PRJNA797736.

## Ethics Statement

The studies involving human participants were reviewed and approved by The Affiliated Drum Tower Hospital of Nanjing University Medical School (ID: 2021-514-02). The patients/participants provided their written informed consent to participate in this study.

## Author Contributions

PY, XZ, RX, XL, MC, and ZX: methodology and data validation. PY: formal analysis. KA provides methodology and language editing. PY and XZ: writing the original draft. HS, ZL, and ZX: conceptualization and supervision. All authors contributed to the article and approved the submitted version.

## Funding

We warmly acknowledge the financial aide provided by the Natural Science Foundation of Jiangsu Province (Grant No. BK20211586), the Qing Lan Project (Grant No. KY101R202127), and the Health Commission Project of Jiangsu Province (Grant No. Z2021004).

## Conflict of Interest

The authors declare that the research was conducted in the absence of any commercial or financial relationships that could be construed as a potential conflict of interest.

## Publisher's Note

All claims expressed in this article are solely those of the authors and do not necessarily represent those of their affiliated organizations, or those of the publisher, the editors and the reviewers. Any product that may be evaluated in this article, or claim that may be made by its manufacturer, is not guaranteed or endorsed by the publisher.

## Abbreviations

GC: gastric cancer
ITS2: internal transcribed spacer region 2
HC: healthy controls
OTUs: operational taxonomic units
AG: atrophic gastritis
IM: intestinal metaplasia
*H. pylori*: Helicobacter pylori
GI: gastrointestinal
LDA: linear discriminant analysis
LEFSe: LDA effect size (LEfSe)
PCoA: Principal Coordinates Analysis
NMDS: nonparametric multidimensional scaling
ROC: receiver operating characteristic
AUC: area under curve
TAMs: Tumor-associated macrophages.
