# Supplementary material for: Fungal Microbiota Dysbiosis and Ecological Alterations in Gastric Cancer
Source: Front Microbiol. 2022 Apr 29;13:889694. doi: 10.3389/fmicb.2022.889694 (PMC9100745; doi:10.3389/fmicb.2022.889694)
Supplement: Supplementary file 1 [file Data_Sheet_1.docx]

**Supplementary Table S1. Primers of immune-related factors**

| **Primer ID** | **Primer sequence** |
| --- | --- |
| FP-*CXCL9* | TTAGCATGCTGGTGAGCCAA |
| RP-*CXCL9* | GCCCTCAAGGAGCTGACAAT |
| FP-*CXCL10* | GCTTCCAAGGATGGACCACA |
| RP-*CXCL10* | GCAGGGTCAGAACATCCACT |
| FP-*CXCL11* | TGTCTTTGCATAGGCCCTGG |
| RP-*CXCL11* | GACTCCTTTGGGCAGTGGAA |
| FP-*TNF-α* | CTGGGCAGGTCTACTTTGGG |
| RP-*TNF-α* | CTGGAGGCCCCAGTTTGAAT |
| FP-*IL-6* | GTCCAGTTGCCTTCTCCCTGG |
| RP-*IL-6* | CCCATGCTACATTTGCCGAAG |
| FP-*CCL17* | ATTCAAAACCAGGGTGTCTCC |
| RP-*CCL17* | CTCTTGTTGTTGGGGTCCGA |
| FP-*IL-10* | AGACAGACTTGCAAAAGAAGGC |
| RP-*IL-10* | TCGAAGCATGTTAGGCAGGTT |
| FP-*YM-1* | CCCTGGACGGAGAGACAAAC |
| RP-*YM-1* | GCCTCAACATGTACCCCACA |
| FP-*IL-4* | CTTTGCTGCCTCCAAGAACAC |
| RP-*IL-4* | GCGAGTGTCCTTCTCATGGT |
| FP-*IL-8* | AGTTTTTGAAGAGGGCTGAGA |
| RP-*IL-8* | ACCAAGGCACAGTGGAACAA |
| FP-*GAPDH* | AGGTCGGTGTGAACGGATTTG |
| RP-*GAPDH* | GGGGTCGTTGATGGCAACA |

**Supplementary Table S2. Clean tags distribution**

| **Distribute** | **Reads** |
| --- | --- |
| 0-200 | 19493 |
| 200-260 | 1125337 |
| 260-320 | 2362046 |
| 320-360 | 452211 |
| 360-380 | 310581 |
| 380-400 | 167983 |
| 400-420 | 10779 |
| 420-440 | 7210 |
| 440-460 | 346 |
| 460-480 | 407 |
| 480-500 | 87 |
| 500-520 | 244 |
| 520-540 | 196 |
| 540-560 | 0 |
| 560-600 | 0 |

**Supplementary Table S3.** **The clinical information of gastric cancer patients**

| **ID** | **Gender** | **Age** | | **Histological type** | **Tumor location** | **Tumor size** | **Pathologic staging** | |
| --- | --- | --- | --- | --- | --- | --- | --- | --- |
| GC1 | Male | 53 | Adenocarcinoma | | Posterior wall of antrum | 3.5cm×3.5cm×1cm | IIIA(T2,N3a,cM0) |  |
| GC2 | Female | 53 | Adenocarcinoma | | Gastric corpus | 3.8cm*3.5cm*1.5cm | IIIA(T4a,N2,cM0) |  |
| GC3 | Male | 37 | Adenocarcinoma | | Antrum | 5cm*4cm*0.5cm | IIIA(T4a,N2,cM0) |  |
| GC4 | Male | 69 | Adenocarcinoma | | Antrum and pylorus | 5cm*4.5cm*1.5cm | IIB(T4a,NO,cM0) |  |
| GC5 | Male | 71 | Adenocarcinoma | | Gastroesophageal junction, lesser curvature | 4cm*3cm*2cm | IIIB(T3,N2,cM0) |  |
| GC6 | Male | 68 | Adenocarcinoma with enteroblastic differentiation | | Gastroesophageal junction, lesser curvature | 6cm*4cm*0.8cm | IIIB(T3,N1,cM0) |  |
| GC7 | Male | 58 | Adenocarcinoma | | Angle of the stomach | 4.3cm*3.2cm*1.5cm | IIB(T4a,NO,M0) |  |
| GC8 | Male | 66 | Adenocarcinoma | | Lesser curvature of the cardia to the posterior wall | 5cm*3cm*1.8cm | IIB(T3,NO,M0) |  |
| GC9 | Male | 57 | Neuroendocrine carcinoma | | Stomach corpus | 8cm×6.5cm×2cm | IIB(T3,N1,cM0) |  |
| GC10 | Female | 82 | Adenocarcinoma | | pylorus | 5.5cm*3.5cm*2cm | IIB(T3,N1,cM0) |  |
| GC11 | Male | 65 | Adenocarcinoma | | Lateral and posterior wall of lesser curvature of cardia | 3.5cm*3cm*1cm | IVA(T4a,N3b,cM0) |  |
| GC12 | Male | 56 | Adenocarcinoma | | Anterior wall of the gastric horns | 4.5cm*3.5cm*0.8cm | IIA(T3,NO,cM0) |  |
| GC13 | Male | 71 | Poorly cohesive type adenocarcinoma | | Posterior wall of gastric corpus | 4cm×3cm×1.5cm | IIIB(T3,N3a,cM0) |  |
| GC14 | Male | 53 | Adenocarcinoma | | Lesser curvature of the gastric corpus | 4cm*3cm*1cm | IIIB(T4a,N3a,cM0) |  |
| GC15 | Male | 67 | Adenocarcinoma | | Lateral to lesser curvature of the cardia | 7cm*6cm*1cm | IIB(T3,N1,M0) |  |
| GC16 | Female | 66 | Poorly differentiated hepatoid adenocarcinoma | | Lateral to lesser curvature of the cardia | 4cm*4cm*1cm | IIIA(T3,N2,cM0) |  |
| GC17 | Female | 45 | Adenocarcinoma | | Greater curvature of the gastric corpus | 9.5cm*3.5cm*0.5cm | IIIC(T4b,N3b,Mx) |  |
| GC18 | Male | 52 | Adenocarcinoma | | Antral lesser curvature side | 4.5cm*4cm*1.5cm | IIIB(T3,N3a,cM0) |  |
| GC19 | Female | 84 | Adenocarcinoma | | Antral lesser curvature side | 4cm*3cm*1cm | IIIB(T3,N3a,cM0) |  |
| GC20 | Male | 55 | Adenocarcinoma | | Lateral to lesser curvature of the cardia | 2cm×1.5cm×1cm | IIB(T3,N0,cM0) |  |
| GC21 | Male | 34 | Adenocarcinoma | | Lateral to lesser curvature of the cardia | 3cm*2cm*1cm | IIIB(ypT3,N2,cM0) |  |
| GC22 | Female | 71 | Adenocarcinoma | | Lateral to lesser curvature of the cardia | 3cm*2cm | IB(ypT2,N0,cM0) |  |


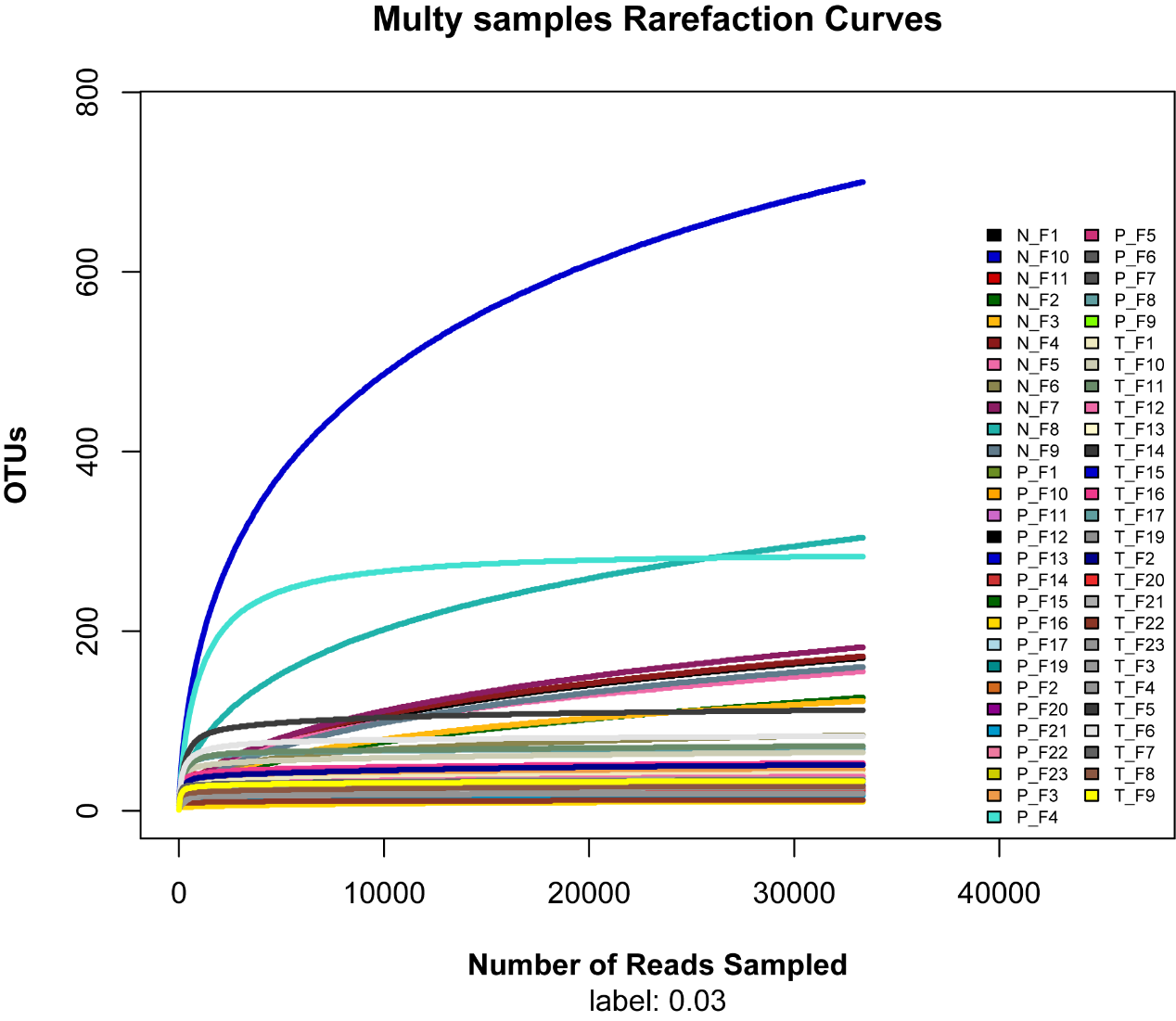


**Supplementary Figure S1. Rarefaction Curves showing the adequate depth of the sequencing of all samples.**

**
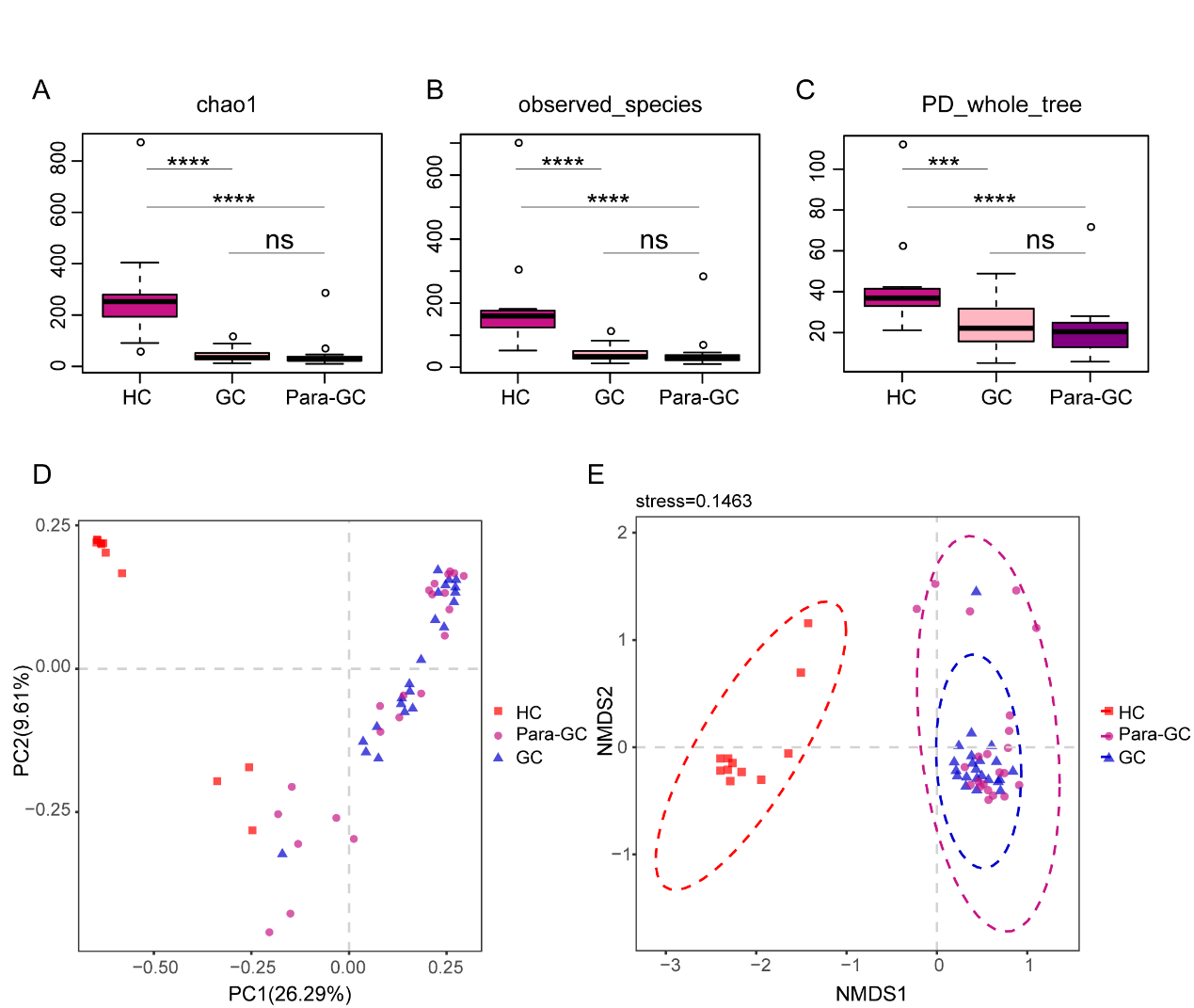
**

**Supplementary Figure S2. Altered bacterial microbiota biodiversity in GC.** (A-C) Alpha diversity. chao1, observed_species, and PD_whole_tree describing the alpha diversity of the fungal in GC, HC, and Para-GC groups (Tukey test, ****p*<0.001; *****p*<0.0001; “ns” means no significance). (D and E) Principal coordinate analysis of Bray–Curtis distance with each sample colored according to different groups. PC1 and PC2 represent the top two principal coordinates that captured most of the diversity. The fraction of diversity captured by the coordinate is given as a percentage. Groups were compared using Permanova method.


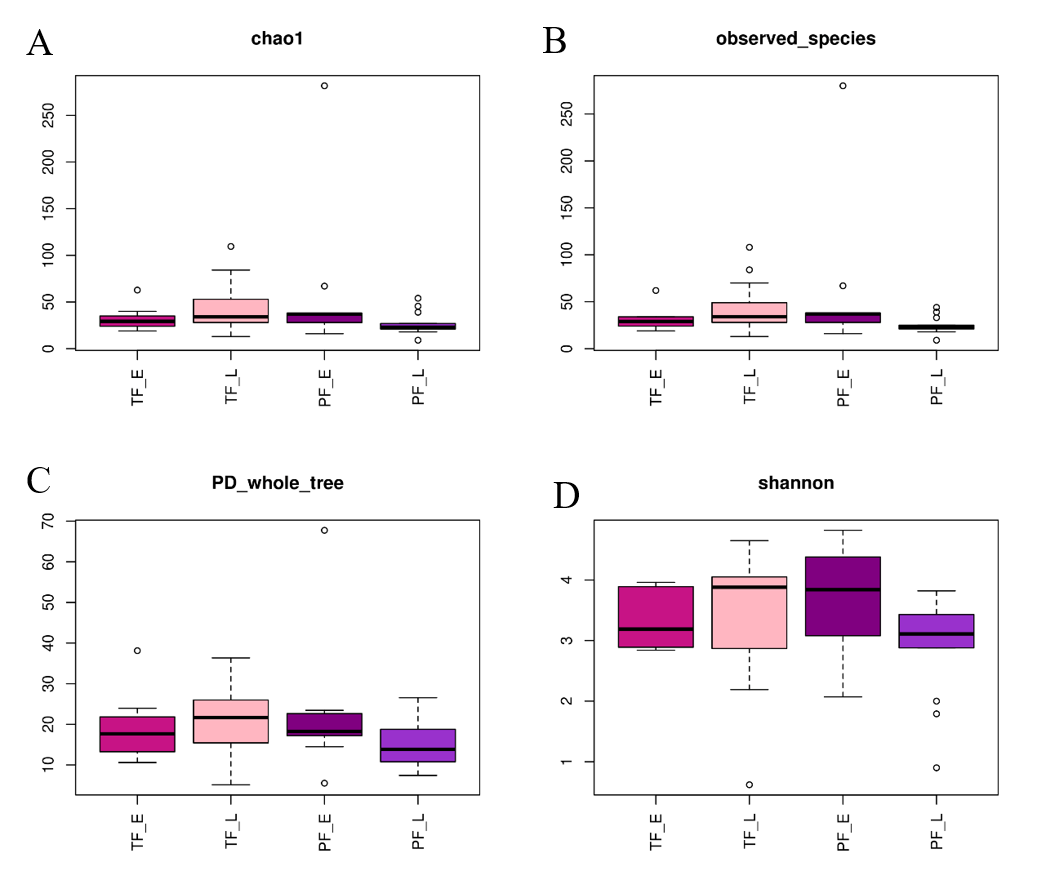


**Supplementary Figure S3.** The alpha diversity of the fungal between early and advanced stage of gastric cancer.

(A-D) chao1, observed_species, PD_whole_tree and shannon describing the alpha diversity of the fungal.

Stages Ⅰ and Ⅱ are defined as early gastric cancer, and stages Ⅲ and Ⅳ are defined as advanced gastric cancer.

**
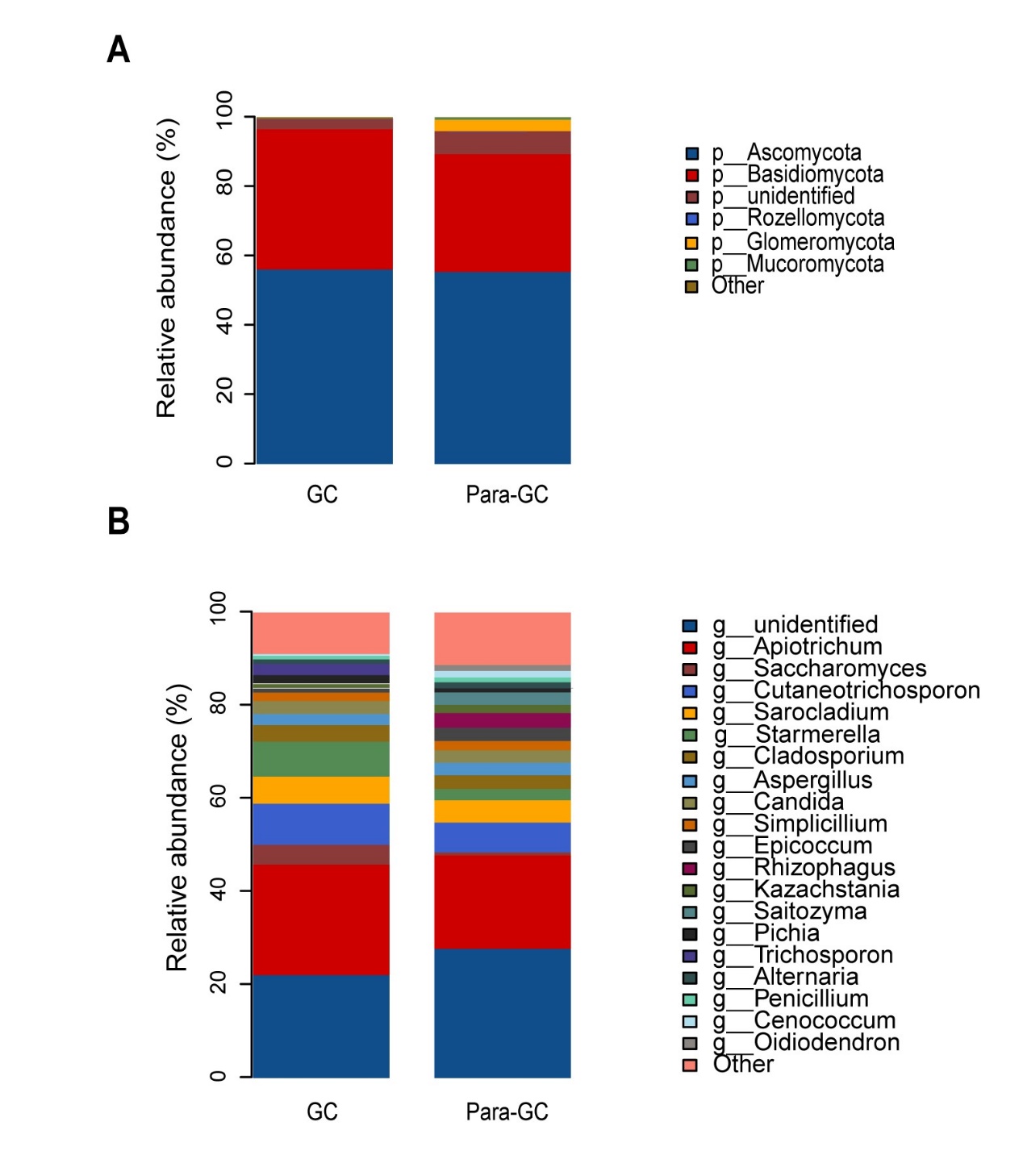
**

**Supplementary Figure S4. Changes in the fungal composition between GC and Para-GC groups.** (A) Comparisons of the relative abundance of dominant fungal taxa at the Phylum level, “p” represents Phylum. (B) Comparisons of the relative abundance of dominant fungal taxa at the Genus level, “g” represents Genus.

**
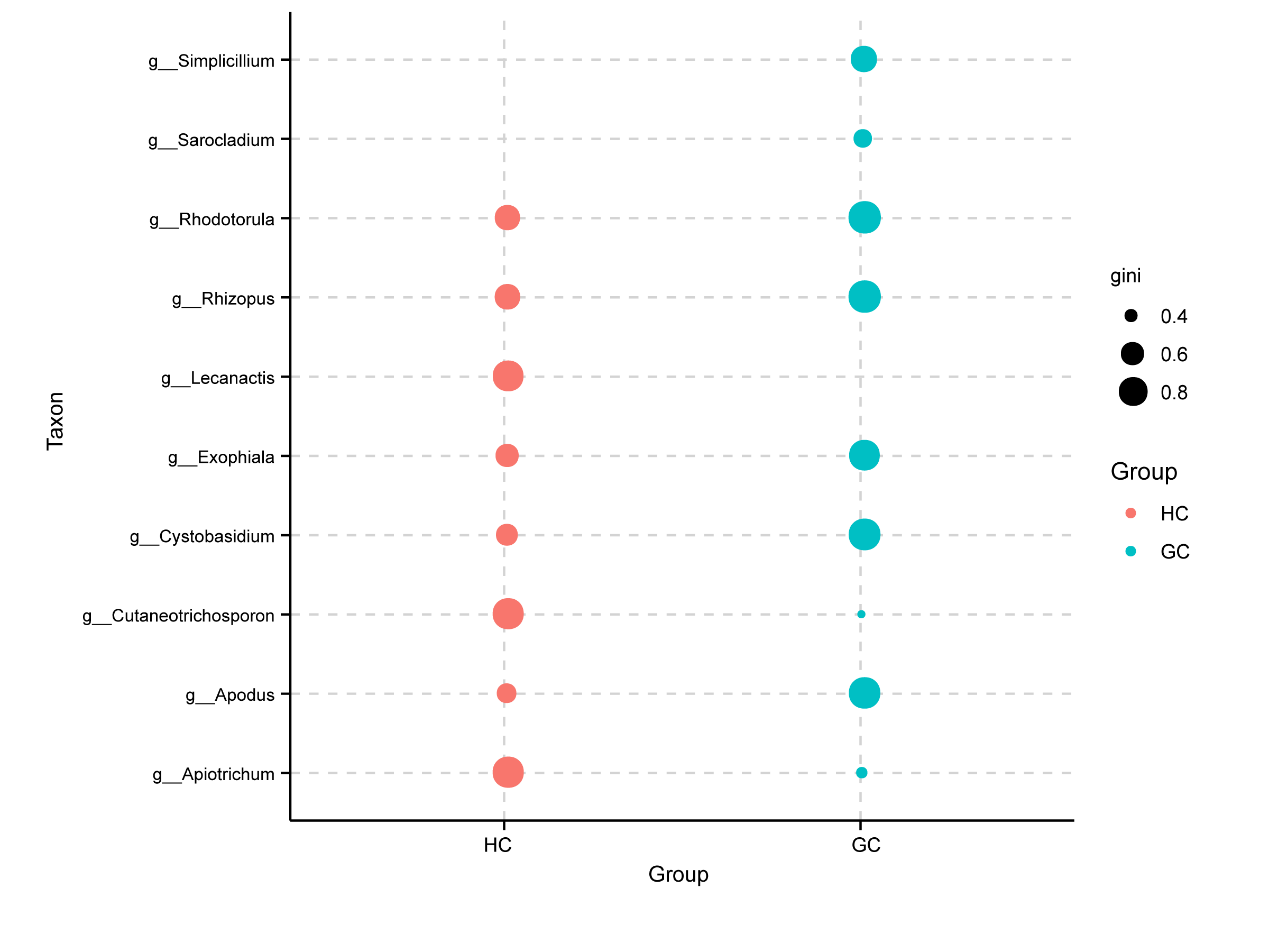
**

**Supplementary Figure S5.** Random forest algorithm showed the contributions of fungi at the genus level to the grouping difference.
